# Influence of Ethylene Oxide and Gamma Irradiation Sterilization Processes on the Properties of Poly-L-Lactic-Acid (PLLA) Materials

**DOI:** 10.3390/polym15163461

**Published:** 2023-08-18

**Authors:** Natalie Krug, Jan-Christoph Zarges, Hans-Peter Heim

**Affiliations:** Institute of Material Engineering, Polymer Engineering, University of Kassel, 34125 Kassel, Germany

**Keywords:** bioplastics, poly-L-lactic-acid (PLLA), sterilization, gamma irradiation, ethylene oxide, single-use products, medical devices

## Abstract

In order to encourage the substitution of petrochemical polymers in medical technology with sustainable, bio-based materials, there is an urgent need for further investigations, especially data regarding their sterility performance. Within the scope of the investigations, selected material properties of poly-L-lactic-acid (PLLA), a specific type of poly(lactic-acid) (PLA), were analyzed before and after sterilization (using ethylene oxide or gamma irradiation) in order to investigate deviations in its chemical structure, wettability, optical, and mechanical properties. In particular, parameters such as molecular weight, complex viscosity, tensile strength, water contact angle, and color were discussed. Sterilization temperatures close to the glass transition of PLA, high humidity, and interactions with the ethylene oxide molecules have resulted in an increase in crystallinity, a decrease in elongation at break, and in some cases, a variation in wettability. As a consequence of exposure to high-energy radiation, the material’s toughness is reduced due to chain scission, which is manifested through a decrease in molecular weight, an increase in crystallinity, and a partial change in surface energy. For the selected PLLA-materials (Luminy^®^ L130, NP HT 202, and NP HT 203), ethylene oxide sterilization resulted in a comparatively minor variation in the characteristics behavior, and was chosen as the preferred method.

## 1. Introduction

The medical and healthcare industry is one of the largest producers of plastic waste in the world. In 2020, the annual plastic waste generated by the healthcare industry in the USA was estimated to be over 1.7 tons [1]. The huge amount of waste can be attributed, among other things, to the high number of single-use items (infusion systems, dialysis catheters, and syringes) used in this sector [1,2]. In the face of climate change and the associated need for a significantly more resource-saving lifestyle, the interest in more sustainable materials is therefore also growing in the field of medical devices.

Technical biopolymers such as the comparatively affordable PLA could be a suitable alternative to petrochemical-based plastics in many different applications [3,4,5,6]. PLA is a linear aliphatic polyester that can be synthesized from only renewable raw materials such as corn or sugar cane [5,7]. Furthermore, PLA is compostable, which reduces the amount of plastic waste. Due to its excellent biocompatibility, PLA is a suitable alternative for medical applications [2,3,4,5,8,9,10,11]. In addition, PLA has been approved by the Food and Drug Administration for contact with food and moreover biological fluids, such as blood, which provides new opportunities for further applications in the healthcare sector [5,9,10,12].

The chiral lactic acid, on which PLA is based, exists either as (L+) lactic or as (D-) lactic. According to the isomer fractions in the material, a distinction can be made between poly-L-lactic (PLLA) and poly-D-lactic (PDLA) [5,12,13]. Specific material properties and, consequently, the exact application areas are significantly dependent on the PLA type. While semi-crystalline PLLA is often used in the area of orthopedics, amorphous PLDA is generally used as a base material for stents [13].

Despite the numerous positive aspects, it is important to note that there is still a lack of knowledge concerning the degradation resistance of the aforementioned resource-saving materials. This knowledge is essential for achieving the substitution of conventional, petrochemical materials. Additionally, in the medical technology sector, it is crucial to highlight the limited availability of data on the sterilization resistance of bioplastics, which play a central role in this industry.

In practice, plastics are usually sterilized using ethylene oxide (EtO) or gamma irradiation (Ga) [4,12,14]. When it comes to applying these two methods to bioplastics, they both exhibit certain disadvantages. In the case of EtO sterilization, the high toxicity of the gas can pose risks to both the operator/patient and the environment. Furthermore, ethylene oxide is highly flammable, carcinogenic, and can cause respiratory problems [12,14,15]. Ga sterilization is a comparatively more expensive process. In addition, irradiation can induce cross-linking as well as the cleavage of chains in the material [10,12,16].

The influence of different sterilization processes on the properties of PLA has been investigated in many studies [3,16,17]. However, the results cannot be summarized into a clear process recommendation. Different types of PLA materials seem to react differently to the stress during sterilization. In this context, the respective parameters of the sterilization process, such as temperature, humidity, irradiation dose, and expansion time also have a notable influence [3,17,18]. In order to bridge this gap in knowledge, this paper focuses on a specific type of PLA, PLLA, and describes its sterilization resistance based on detailed mechanical, thermal, optical, and chemical investigations. Therefore, this work aims to improve the scientific understanding of the material behavior of PLLA in course of the two most established sterilization methods. Consequently, it provides an enhanced knowledge for the estimation of the shelf life and the prediction of the aging behavior.

## 2. Experimental

In this work, the properties of three PLLA types (Luminy^®^ L130, NP HT 202, and NP HT 203) were analyzed using gel permeation chromatography, rheometric analysis, Fourier transform infrared spectrometry, discoloration analysis, tensile tests, impact tests, and contact angle measurements before and after sterilization. Afterward, a direct comparison of the determined parameters was conducted. All the materials used are described as comparatively resistant to high temperatures by the respective manufacturers. Sterilization of all materials was carried out using ethylene oxide gas or gamma irradiation. The parameters used were exactly the same for all materials.

### 2.1. Materials

Three different types of PLLA were used in this study. NP HT 202 and NP HT 203 were distributed by NaturePlast (Mondeville, France) and Luminy^®^ L130 was supplied by TotalEnergies Corbion (Gorinchem, The Netherlands). All used materials have been certified for contact with food and can be processed through injection molding. Their characteristic properties are shown in Table 1. No additives were used.

### 2.2. Injection Molding Process

All materials were stored in a dry and dark place in sealed bags. Since the PLA has hydrophilic characteristics, it was dried for 4 h at 100 °C (Luminy^®^ L130) or 60 °C (NP HT 202, NP HT 203) using a Vacucenter VC 50 vacuum oven (Salvislab, Risch-Rotkreuz, Switzerland) prior to processing. The dried material was used to produce standardized 1A test specimens (DIN EN ISO 527 [19]) through injection molding, employing an Arburg Allrounder 270S injection molding machine (Arburg GmbH + Co KG, Loβburg, Germany) with a clamping force of 25 kN and a screw diameter of 22 mm.

For Luminy^®^ L130, a cycle time of 50.8 s and a cooling time of 40 s were used. NP HT 202 was processed with a cycle time of 62.4 s and a cooling time of 50 s. The comparatively shortest cycle time (46.1 s) was used with NP HT 203. The corresponding cooling time was 2.7 s. The temperature profile of the injection molding processes is shown in Table 2. The entire injection molding process was conducted without the use of a release agent, regardless of the material.

### 2.3. Packaging

For further investigations regarding storage and sterilization, 15 test specimens of the same material were packed together in one low-density polyethylene bag for storage and/or Ga-sterilization, or a peel bag (CarePrime, Voerde, Germany) for EtO-sterilization. Only the test specimens of Luminy^®^ L130 were individually packaged to prevent the samples from sticking to each other during annealing (Section 2.4). Additionally, 80 g of non-dried granulate from each material was packed in PE-LD bags or peel bags. Due to the use of non-processed granulate when analyzing viscosity and molecular weight, it can be ensured that the measurement results were not influenced by material damage during sample comminution.

### 2.4. Annealing

Due to the slow crystallization kinetics of PLA, samples obtained by the typical injection molding process are usually amorphous [20]. The 1A test specimens and granulate of Luminy^®^ L130 were stored for 2 h at 80 °C in an air convection oven manufactured by Memmert (Schwabach, Germany) to enhance the initial crystallinity and, consequently, the heat deflection temperature. In this context, thermal pre-treatment also aids in stabilizing the material, especially concerning artificially accelerated aging [14,18]. NP HT 202 and NP HT 203 are classified as materials with high heat distortion resistance, even in the amorphous state; therefore, annealing was not necessary.

### 2.5. Sterilization

Test specimens and granulate were sterilized using either ethylene oxide gas or gamma irradiation and subsequently compared with a non-sterile reference. Sterilization was performed by a medical technology company (B. Braun, Melsungen AG, Melsungen, Germany).

During the sterilization process with EtO, the highly reactive, toxic gas comes into contact with germs and microorganisms. As a result, alkylation reactions caused by this lead to the denaturation of nucleic acids and functional protein as well as enzymes. Specifically, cell metabolism is inhibited by the adhesion of alkyl groups to sulfhydryl, hydroxyl, carboxyl, and amino groups. The organisms lose their viability [12,21,22]. For better EtO diffusivity this process requires extremely high humidity and temperatures in the range of the glass transition temperature of PLA [12,21].

Due to sterilization with Ga irradiation, in the form of short electromagnetic wavelengths, the absorption of photon energy within the material leads to the formation of high-energy electrons, which in turn cause the ionization of the material. From the resulting primary electrons, secondary electrons and finally free radicals are produced [23]. All in all, sterilization with Ga radiation leads to an ionization reaction of the amino acids in the cell nucleus of the microorganisms, and thus to the splitting of the DNA due to free radicals [23,24]. The cells lose their ability to multiply and die [12,25]. Medical products are usually sterilized at an irradiation dose of 25 to 30 kGy [6,23,24]. Within the scope of this paper, the samples were exposed to an irradiation dose of 45 kGy to maximize the impact.

Due to confidentiality reasons, the exact process parameters of both sterilization processes are not disclosed.

### 2.6. Characterization

After sterilization, all samples were conditioned at the standard climate of 23 °C and 50% relative humidity for at least 24 h before conducting mechanical characterization. Storage of samples and granules between experiments was also maintained under these controlled environmental conditions to limit climate-induced alterations in material properties.

#### 2.6.1. Gel Permeation Chromatography (GPC)

The number average molecular weight (Mn), the weight average molecular weight (Mw), and the polydispersity (D) were determined with help of gel permeation chromatography. Therefore, chloroform was used as both the solvent and eluent agent. The flow rate was set to 0.75 mL/min and the measuring temperature was defined as 30 °C. As a precaution, the calibration was carried out with a polystyrene standard with a narrow distribution. For this characterization method, granulated samples were used.

#### 2.6.2. Parallel-Plate Rheometry

For shear rheological characterization, the dynamic viscosity (η*) was investigated using granulate samples. A DHR 2 Discovery Hybrid rheometer (TA Instruments, Eschborn, Germany) with a 25 mm parallel-plate geometry was used for each analysis. Both amplitude and frequency sweeps at 190 °C were performed with the samples. With the frequency sweeps, the sample was melted between the two plates of the rheometer, and angular frequencies from 0.01 to 630 rad/s were applied to the upper plate.

#### 2.6.3. Fourier Transform Infrared Spectroscopy (FT-IR)

Attenuated total reflectance FT-IR was applied for the identification and characterization of materials produced during the degradation process. For this purpose, measurements were performed in the wavelength segment from wavenumber 600 cm^−1^ to 4000 cm^−1^ using an IRAffinity-1S by Shimadzu (Duisburg, Germany) and a zinc–selenium crystal. The measurement resolution was set to 2 cm^−1^. All measurements were carried out in the shoulder area of the test specimens to detect chemical modifications.

#### 2.6.4. Color Measurement in the CIELAB Space

Color characteristics were identified based on the specifications from the Commission Internationale de l’Eclairage (CIE). Therefore, the CIELAB system was used to determine the three components of color (L* luminosity; a* red-green; b* yellow-blue). Color difference, ∆E, was measured using an Ultra Scan Pro (Hunterlab, Murnau, Germany). The measurements were performed in the parallel area of the test specimens. A total of five specimens per group were analyzed. Classification was conducted using the color difference ∆E, which is determined using Equation (1).

(1) $$\Delta E=\sqrt{{\Delta L}^{2}+{\Delta a}^{2}+{\Delta b}^{2}}$$

#### 2.6.5. Tensile Test

To determine Young’s modulus, tensile strength, and elongation at break, a Zwick/Roell Z010 Universal Testing Machine (Ulm, Germany) was used to perform tensile tests with 1A test specimens along the longitudinal axis according to DIN EN ISO 527. The tests were performed with a pre-load of 0.1 MPa and a crosshead speed of 5 mm/min (Luminy^®^ L130; NP HT 202) or 25 mm/min (NP HT 203). Five specimens were tested for each condition.

#### 2.6.6. Notched Impact Test (According to Charpy)

Instrumented notched Charpy impact tests were applied in order to determine the impact strength according to DIN EN ISO 179 [26] with a Zwick Charpy pendulum (Ulm, Germany) using a 5 J hammer. These tests were performed in a climate-controlled environment at 23 °C and 50% relative humidity. Notched specimens (type A) with a size of 80 mm × 10 mm × 4 mm were cut out of 1A test specimens. Ten specimens were tested for each group.

#### 2.6.7. Contact Angle Measurement

Water contact angle (α) measurement was assessed by the method of sessile drop (volume of 1–2 µL) with distilled water. The tests were carried out with the Drop Shape Analyses (DSA) 20B from Krüss (Hamburg, Germany) in an environment with a temperature of approximately 20 °C. Ten drops on two specimens of each condition were observed, which were applied in the parallel section of the tensile test specimen.

## 3. Results and Discussion

During storage and annealing, Luminy^®^ L130 specimens exhibited notable deformations. As a result of these deformations, not all tests could be carried out using specimens compliant with standards. This circumstance led to higher standard deviations, especially in the mechanical properties.

### 3.1. GPC Analysis

**Figure 1:**
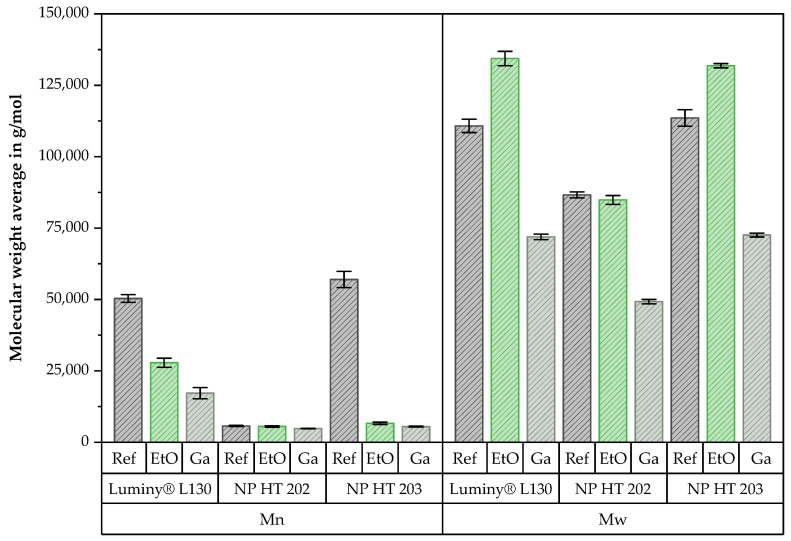
Number average molecular weight *Mn* and weight average molecular weight *Mw* of Luminy^®^ L130, NP HT 202, and NP HT 203 before and after sterilization by EtO or Ga; *n* = 3.

In contrast to Luminy^®^ L130, NP HT 203 and NP HT 202 were not entirely soluble in chloroform, which suggests the presence of an additive in these materials. Nevertheless, similar changes as a result of sterilization were documented for all three materials. Figure 1 exhibits the average molecular weight measured with GPC for Luminy^®^ L130, NP HT 202, and NP HT 203.

The reference of the Luminy^®^ L130 and NP HT 203 only slightly differed in Mw with 1.11 × 10^5^ g/mol (Luminy^®^ L130) and 1.14 × 10^5^ g/mol (NP HT 203). In comparison, a lower value (8.66 × 10^4^ g/mol) was measured for NP HT 202. As the GPC analysis of this material was conducted approximately two months later, it is possible that these differences can be attributed to advanced aging. Regarding Mn, the differences were relatively more significant. The molecular weights (Mn) of Luminy^®^ L130, NP HT 202, and NP HT 203 were reported as values of 5.03 × 10^4^ g/mol, 5.7 × 10^3^ g/mol, and 5.70 × 10^4^ g/mol, respectively.

The results presented in Figure 1 indicate that sterilization with ethylene oxide results in a broader molecular weight distribution (polydispersity). This assumption is based on increasing Mw (Luminy^®^ L130: +21%; NP HT 203: +16%) while decreasing Mn (Luminy^®^ L130: −0.45%; NP HT 203: −88%). As indicated by the results of NP HT 202 and additional supplementary measurements, which are not explicitly listed here, the difference, and thus the polydispersity index, appears to become more uniform over the course of aging.

The reduction in Mw and Mn can be a result of hydrolysis that occurs during EtO sterilization [3,4,13,27,28]. Because of the reduced Mn, it can be assumed that hydrolysis also occurred during the EtO sterilization in the context of this work. In contrast to that, the increase in Mw could be explained by possible simultaneous cross-linking reactions between the ethylene oxide molecules and the hydroxyl and/or carboxyl groups of the polymer [4].

The chain length as well as the molecular weight of the polymer molecules (Mw and Mn) were significantly reduced by Ga sterilization. This observation is supported by Sternberg and others [4,6,10,14,18,28,29,30,31]. The decrease in molecular weight is attributed to the high energy input, leading to radical formation and subsequent chain scission, particularly in the amorphous regions of the polymer [10,16,18]. As a result of the higher oxygen diffusivity of these areas, the reaction of oxygen with alkyl free radicals is encouraged. In this process, peroxyl free radicals are formed, which in turn, cause chain scission through hydrogen abstraction [10,18]. The reduced molecular mass, particularly when combined with the higher polydispersity index, as documented in these investigations, is attributed to the degradation [10,18]. A strong correlation between the irradiation dose and the reduction of the molecular weight was described in the following studies [6,10,16,31]. Moreover, this connection was also documented by Aouat et al. and others for gamma-irradiation-induced crosslinking, which is enhanced at doses above 250 kGy [16]. The results generated in this study also do not suggest a dominant crosslinking effect at the used dose of 45 kGy.

### 3.2. Complex Viscosity

**Figure 2:**
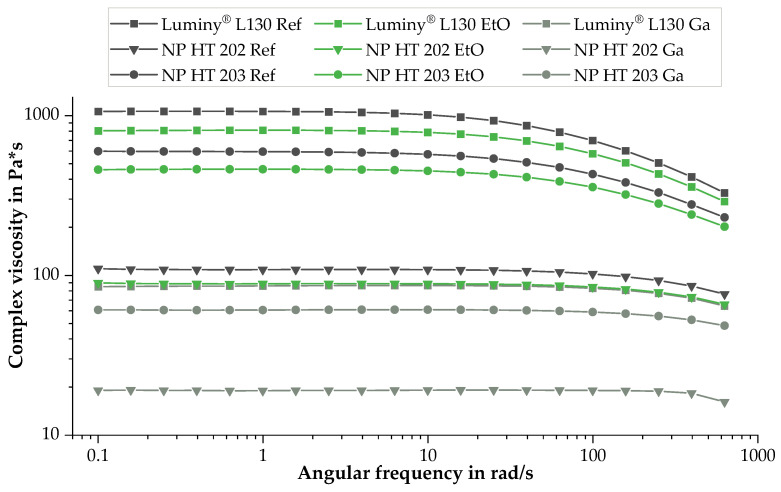
Complex viscosity η* of Luminy^®^ L130, NP HT 202, and NP HT 203 at 190 °C before and after sterilization by EtO or Ga; *n* = 1.

The analysis of the degradation behavior during sterilization was enhanced by further investigations using a rotational parallel-plate rheometer. Compared to GPC, this method allows us to examine the shear viscous performance over a wide frequency range. Figure 2 reveals the effect of the sterilization process by plotting the complex viscosity over the angular frequency.

The characterized materials exhibit typical thermoplastic viscosity distributions, indicating a structurally viscous fluidity. Nevertheless, there were noticeable differences in the initial viscosity among the analyzed PLLA types. The Newtonian plateaus of Luminy^®^ L130 and NP HT 203 were found to be 1063 Pa*s and 596 Pa*s, whereas the zero viscosity of NP HT 202 was reported as 108 Pa*s. Furthermore, it can be observed that sterilization resulted in a decrease in zero viscosity, indicating polymer chain degradation for all tested materials. The shortened polymer chains can unwind and orient more easily under shear deformation, leading to reduced resistance against flow and, consequently, a decreased viscosity. A relatively higher reduction due to Ga sterilization can be observed here, which is consistent with the results of Mn illustrated in Figure 1.

Regardless of the material, the complex viscosity was reduced by EtO sterilization. This result supports the assumption that hydrolytic cleavage of polymer chains occurred during EtO sterilization. A relative decrease of 24% was measured for Luminy^®^ L130. NP HT 202 showed a 23% reduction in viscosity as a result of EtO sterilization, and for NP HT 203 −19% was recorded. Whether the cross-linking reactions mentioned in Section 3.1 also occurred cannot be assessed from these results. In the context of this work, the applied radiation dose led to a decrease in viscosity of at least 80%. The largest reduction (−91%) was observed with Luminy^®^ L130. A relative decrease of 90% and 82% was determined for NP HT 202 and NP HT 203, respectively.

### 3.3. FT-IR Analysis

**Figure 3:**
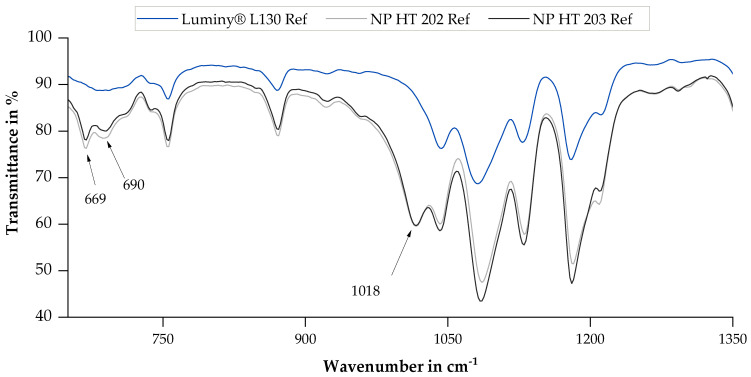
FT-IR transmission spectrum of the reference samples of all examined materials after baseline and atmosphere correction.

In connection with the FT-IR analysis, each measurement spectrum revealed absorption bands at 1044 cm^−1^ ($v\left(C-CH_{3}\right))$, 1183 cm^−1^ $\left(v\left(C-O-C\right)+r_{as}\left(CH_{3}\right)\right)$, and 1749 cm^−1^ $\left(\left(v\left(C=O\right)\right)\right)$ which are characteristic for pure PLA [3,16,32]. A comparison of the reference samples of all examined materials, which can be seen in Figure 3, shows that both NP HT 202 and NP HT 203 create two additional transmittance peaks at wavenumbers of 669 cm^−1^, 690 cm^−1^, and 1018 cm^−1^, respectively, compared to Luminy^®^ L130. These peaks are attributed to additives present in the material, which have already been identified during GPC sample preparation (as mentioned in Section 3.1).

Based on the generated FT-IR spectra, apparent changes in the chemical composition of the materials due to sterilization can be ruled out. Regardless of the PLLA type, no disappearance or appearance of peaks was documented when comparing the spectra of sterile and non-sterilized specimen. This observation is consistent with the results of Hooper et al. [27]. Nevertheless, intensities that differed significantly from each other were documented in some cases, as described below.

According to Savaris et al., an increased absorption in the range of 3000 to 3500 cm^−1^ indicates hydrolytic degradation following EtO sterilization [17]. More precisely, peaks in this range can be traced back to terminal OH groups [33]. In the context of this work, a similar effect was also observed in NP HT 202. However, for Luminy^®^ L130 and NP HT 203, no peaks were documented in this range, which could be attributed to the method’s insufficient sensitivity. This notion is further supported by the fact that the FT-IR analysis was not able to detect any additional modifications resulting from EtO sterilization within the scope of this study.

**Figure 4:**
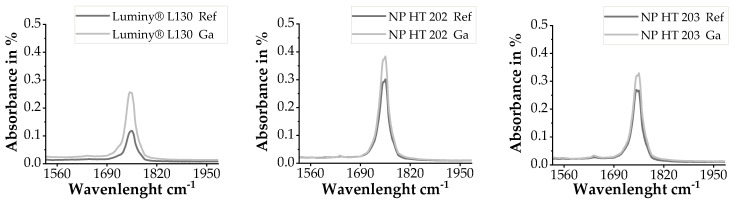
FT-IR absorbance spectrum of the reference and Ga-sterilized samples of all examined materials after baseline and atmosphere correction.

An increase in the crystallinity due to Ga sterilization can be inferred due to the reduction in transmission at a wavenumber of 860 cm^−1^ and 956 cm^−1^. These wavenumbers are assigned to the amorphous fraction of PLA [3,32]. The increase in absorption at 755 cm^−1^ and 872 cm^−1^, referring to the crystalline alpha phases, confirms this assumption [32]. Aouat et al. described a significant increase in absorption at 1745 cm^−1^ as a result of the Ga sterilization of PLA, which can be attributed to stretching of C=O in the material that results in chain scission [16,33]. In other studies, this extension is related to PLA degradation [33]. As can be seen in Figure 4, an increase at 1745 cm^−1^ was also documented for Ga-sterilized samples in the present study.

### 3.4. Color

**Figure 5:**
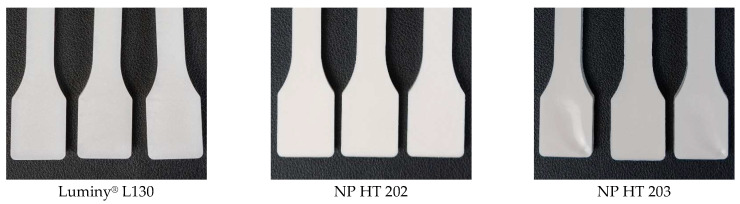
Comparison of specimen color by shoulder area of sterile and non-sterilized specimens. From left to right: Ref, EtO, Ga.

Within the scope of these examinations, no discoloration of the test specimens was visible to the naked eye as a result of sterilization. In order to illustrate this, the test specimens of each material before and after sterilization are shown in Figure 5.

**Figure 6:**
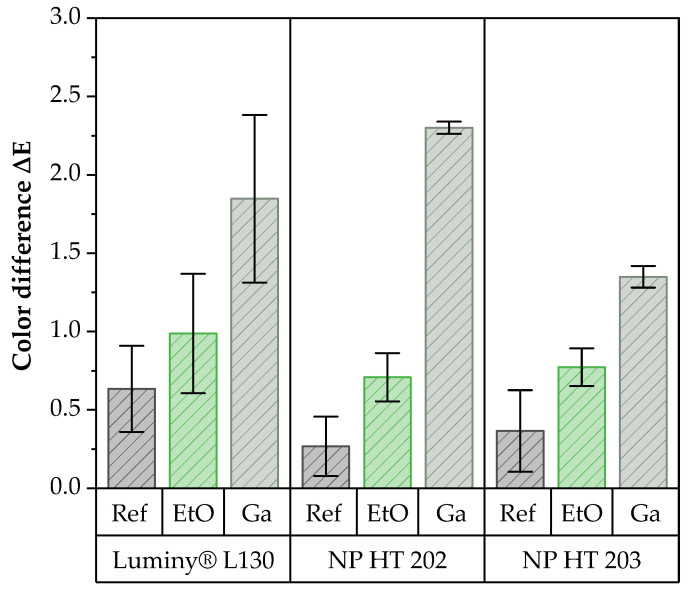
Color difference ∆E of the test specimens in the CIELAB space before and after sterilization with EtO or Ga. A minimum sample size of *n* = 5 was used.

The computer-assisted examinations listed in Figure 6 confirm this result. Color changes were visualized based on the color difference. This was calculated from the total of the color deviations in the CIELAB space and thus provides a coefficient for total color variation.

The documented changes displayed a color difference of less than 3 in each case, which can be classified as negligible [34]. These results are consistent with the data from the literature [3,10,17,27].

The results showed that the material became slightly darker (L*: −0.86) and turned blue (b*: −0.52) all along the EtO sterilization. Through Ga sterilization, the whitish material was also darker (L*: −2.03) and slightly yellower (b*: +0.9) afterwards. The shade of NP HT 202 was not changed by the EtO sterilization. In contrast to this, NP HT 202 also showed a slightly darker color (L*: −2.17) because of Ga sterilization. In addition, the samples became bluer (b*: −0.99), but the a* values did not change. NP HT 203, once again, showed no change in color after EtO sterilization. As a result of Ga sterilization, the surface became darker (L*: −1.39) and slightly more blue (b*: −0.54).

Furthermore, yellowish discoloration of polymeric materials was often observed following Ga sterilization, which is explained in the literature as the formation of secondary alkyl radicals, which are formed through the release of H2 from the separation of C-H bonds as a result of the irradiation [17,23,35]. As mentioned before, no yellowing or only very slight yellowing was observed in the course of this work. Consequently, the slight color changes could be attributed to the shift in the ratio between amorphous and crystalline fractions in the material, which causes a change in the refractive index (transparency) [17]. With reference to this correlation and the results given in Section 3.3, the comparatively higher discoloration due to Ga sterilization can be attributed to a stronger increase in crystallinity.

### 3.5. Mechanical Properties

**Figure 7:**
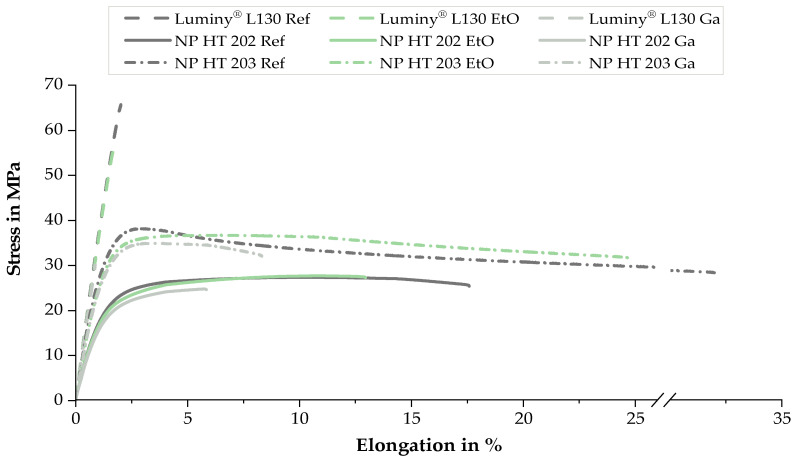
Representative stress–strain curves of all tested materials before and after sterilization. The tests were performed with a pre-load of 0.1 MPa and a crosshead speed of 5 mm/min (Luminy^®^ L130; NP HT 202) or 25 mm/min (NP HT 203).

**Figure 8:**
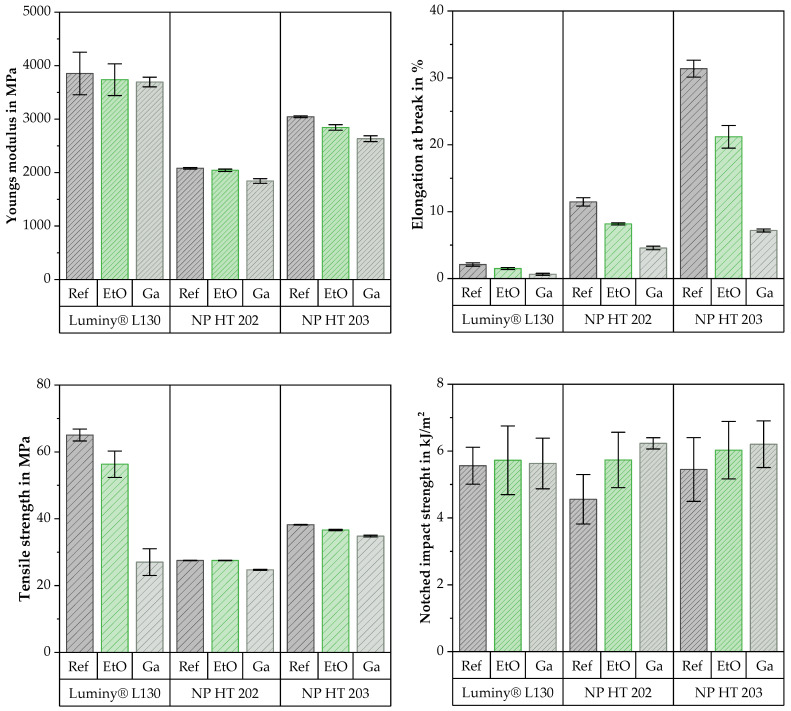
Difference in mechanical properties as a result of EtO or Ga sterilization in relation to the non-sterile, material-specific initial value.

In terms of mechanical properties, similar trends were observed during the tensile test for all tested materials as a result of sterilization. These results allow for the derivation of uniform trends for the analyzed materials. Figure 7 illustrates representative stress–strain curves of the materials before and after sterilization. Exact values are shown in Figure 8 using tensile strength, elongation at break, and Young’s modulus. In addition the Charpy notched impact strength is also shown in Figure 8.

In the context of this work, sterilization with ethylene oxide resulted in a reduction in elongation at break and a slight increase in notched impact strength for all the tested materials. Concerning elongation at break, a connection between the initial value and the intensity of the absolute reduction was identified. The largest absolute difference was observed in the material with the highest initial elongation at break. NP HT 203 showed a 10% reduction as a result of EtO sterilization, while NP HT 202 and Luminy^®^ L130 exhibited absolute reductions of 3.3% and 0.6%, respectively. Peniston et al. attributed the reduced elasticity to the molecular entanglements associated with degradation [4]. This correlation can also be utilized to explain the slight increase in notched impact strength that tends to occur as a result of EtO sterilization.

Consistent with Zhao et al. and other studies, the Young’s modulus and tensile strength were either not changed or changed only to a very small extent [3,4,27]. Only Luminy^®^ L130 showed a clear reduction in tensile strength of 9 MPa after EtO sterilization.

In the scope of these investigations, sterilization with gamma irradiation led to a reduction in tensile strength, Young’s modulus, and elongation at break. This observation is in line with the literature and can be attributed to the predominance of random chain scissions rather than crosslinking [10,14,16,35,36]. In the context of this work, it is important to mention that there are clear differences in the intensity of the material change.

The material with the lowest MFI (Luminy^®^ L130) experienced a significant reduction in elongation at break (−70%) and the highest decrease in tensile strength (−58%) due to irradiation. In comparison with the other materials, Young’s modulus, which decreased by 4%, was the least affected. Overall, the mechanical properties of NP HT 202, the material with the highest initial MFI, comparatively experienced the least alterations after Ga sterilization. The tensile strength and elongation at break were lowered by 10 and 60%, respectively, while Young′s modulus was reduced by 11%. NP HT 203 showed the most significant decrease in Young’s modulus (−13%) and elongation at break (−77%). The tensile strength was reduced by 9%. In evaluating these results, the higher test speed used should be considered.

Regardless of the sterilization method, the notched impact strength of Luminy^®^ L130 was not changed by sterilization in the course of these investigations. Each of the three groups of specimens deviated to a greater or lesser extent around the mean value of the reference group, which can be given as 5.56 kJ/m^2^. From the results of NP HT 202 and NP HT 203, it can be suspected that the notched impact strength was increased by sterilization.

The average notched impact strength of NP HT 202 was increased by 1.18 kJ/m^2^ as a result of EtO sterilization. However, this deviation is not considered significant due to a *p*-value of 0.6. This contrasts with the significant increase (*p* = 0.2) due to Ga sterilization, which can be given as 1.67 kJ/m^2^ on average. The average notched impact strength of NP HT 203 was increased by 0.58 kJ/m^2^ (EtO) and 0.76 kJ/m^2^. Using the calculated *p*-values (0.25 for EtO and 0.17 for Ga), the deviation is not considered to be significant under both methods.

### 3.6. Water Contact Angle

**Figure 9:**
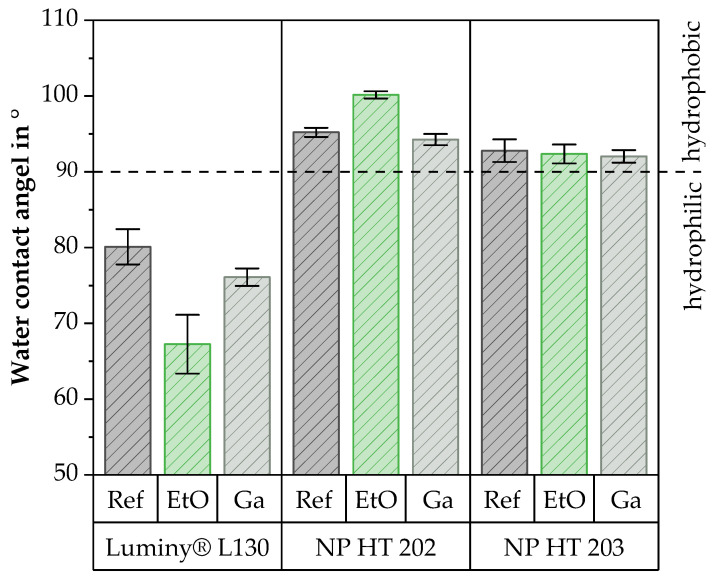
Water contact angle of all materials before and after sterilization by EtO or Ga sterilization.

Luminy^®^ L130 showed a hydrophilic character, which is usual for PLA. Conversely, hydrophilic surfaces were documented for NP HT 202 and NP HT 203, irrespective of the condition. Figure 9 illustrates the results of the measurements.

As can be seen, the water contact angle of Luminy^®^ L130 was lowered by EtO sterilization. This change is due to the polar cleavage products (lactic acid), which are formed during the hydrolysis of PLLA by the cleavage of the ester bonds. The increase in polarity increases the wettability with water and reduces the contact angle. These observations are consistent with the literature, which also documented a slight increase in the water contact angle at room temperature as a result of EtO sterilization [4,15]. Bednarz et al. [15] suspect that the reduction in wettability is due to the change in surface free energy resulting from the radical reaction of the EtO molecule with the ends of the PLA chain [15]. Savaris et al., in turn, attribute the increase in wettability to the absorption of water into the outer layers of the material. The surface thus becomes more hydrophilic [17].

Despite the fact that NP HT 202 and NP HT 203 are also PLA, they showed a slight decrease in wettability and an increase in the water contact angle as a result of EtO sterilization. This observation could be explained by additives in the material, so-called radical scavengers, which catch the polar cleavage products. The existence of the additives was also indicated in the GPC and FT-IR analyses.

In contrast, the water contact angles of all analyzed materials were slightly reduced by Ga sterilization. In this context, Luminy^®^ L130 showed the comparatively highest reduction in the water contact angle. The literature also documents minor to negligible changes in the water contact angle as a result of Ga sterilization [17,37].

## 4. Conclusions

EtO sterilization led to a decrease in viscosity and elongation at break as well as an increase in crystallinity and notched impact strength for all tested materials. One of the factors that determine the extent of the change was the initial reference value. Differences in color were not observed in the initial white or opaque materials. Overall, the materials examined were classified as suitable for EtO sterilization. Furthermore, regardless of the sterilized type of material, this method should be questioned due to the high toxicity of the gas and the associated ecological damage [14,15].

As a result of Ga sterilization, a clear degradation of the material was observed, which was reflected by the embrittlement. During the course of this work, this is shown by a decrease in molecular weight, viscosity, elongation at break, and tensile strength. Although the crystallinity and notched impact strength increased, the color did not change. Several studies indicated that material degradation intensity is primarily related to the irradiation dose [31]. In this context, it should be mentioned that the used irradiation dose of 45 kGy was deliberately chosen to be significantly higher than the actual average value [14]. Therefore, it cannot be ruled out that the material damage would be less in actual circumstances. Treatment of the examined materials with the irradiation dose used here is not recommended. With regard to medical applications, it should also be noted that PLLA with a low molecular weight is suspected of causing inflammation and in-stent restenosis, which also indicates that this method should be avoided [28].

Additionally, it should be noted that Ga sterilization, due to its high energy input, can result in an increase in the material temperature (according to Harrington et al., 30–40 °C within a few hours) [23]. This work did not examine the extent to which the documented material damage can be attributed to this effect. However, this connection could be a useful approach for further investigations.

These investigations have shown that PLLA is altered by sterilization with Ga as well as with EtO. A direct comparison leads to a preference for sterilization with EtO for the materials examined here. When classifying these data, it is essential to acknowledge that the results were significantly influenced by the exposure during sterilization, the specific composition of the material, and the age of the sample.

## Figures and Tables

**Table 1:** Density, melt-flow index (MFI), bio-based mass content, and heat deflection temperature (HDT) of used materials. The listed values correspond to the manufacturer’s information.

Material	Density in g/cm^3^	MFI 190 °C/2.16 kg in g/10 min	Bio-Based Mass Content in %	HDT in °C
Luminy^®^ L130	1.24	10 *	100	105 **
NP HT 202	1.33	39 *	85	120
NP HT 203	1.24	17–23	90	118

* Own measurements deviate significantly from this value; ** crystalline.

**Table 2:** Temperature profiles used in the processing of the selected materials.

Zone	Material	1 Feeding Section	2	3	4 Nozzle	Mold
Temperature in °C	Luminy^®^ L130	205	205	215	215	40
	NP HT 202	175	180	185	185	25
	NP HT 203	175	180	185	185	25

## Data Availability

The data presented in this study are available on request from the corresponding author.
